# A case of swine influenza A(H1N2)v in England, November 2023

**DOI:** 10.2807/1560-7917.ES.2024.29.3.2400002

**Published:** 2024-01-18

**Authors:** Jade Cogdale, Beatrix Kele, Richard Myers, Ruth Harvey, Abi Lofts, Tanya Mikaiel, Katja Hoschler, Ashley C Banyard, Joe James, Benjamin C Mollett, Alexander MP Byrne, Jamie Lopez-Bernal, Conall H Watson, Meera Chand, William Welfare, Deborah A Williamson, Isabel Oliver, Simon Padfield, Andrew Lee, Suzanne Calvert, Martin A Bewley, Louise Wallace, Simon deLusignan, Nicola S Lewis, Ian H Brown, Maria Zambon, on behalf of the Influenza A(H1N2)v Incident Management Team

**Affiliations:** 1 United Kingdom Health Security Agency (UKHSA), London, United Kingdom; 2 The Francis Crick Institute, London, United Kingdom; 3 Animal and Plant Health Agency (APHA), Weybridge, United Kingdom; 4 Royal Veterinary College, London, United Kingdom; 5 United Kingdom Health Security Agency (UKHSA), Manchester, United Kingdom; 6 United Kingdom Health Security Agency (UKHSA), Leeds, United Kingdom; 7 Yorkshire and Humber Public Health Network (YHPHN), Yorkshire, United Kingdom; 8 Nuffield Department of Primary Care Health Sciences, University of Oxford, Oxford United Kingdom; 9 Royal College of General Practice (RCGP) Research and Surveillance Centre (RSC), Oxford, United Kingdom

**Keywords:** Influenza A, Swine, H1N2, Zoonosis, Surveillance, England

## Abstract

Under International Health Regulations from 2005, a human infection caused by a novel influenza A virus variant is considered an event that has potential for high public health impact and is immediately notifiable to the World Health Organisation. We here describe the clinical, epidemiological and virological features of a confirmed human case of swine influenza A(H1N2)v in England detected through community respiratory virus surveillance. Swabbing and contact tracing helped refine public health risk assessment, following this unusual and unexpected finding.

Influenza viruses circulating in swine are genetically diverse. These animals are susceptible to porcine, human and avian influenza viruses, providing the opportunity for generation of novel viruses arising from mixing of viral gene segments when co-infections occur. Variant viruses may be transmitted to humans through sporadic zoonotic infection events, some of which may pose a significant pandemic threat if there is sustained human-to-human transmission, such as happened in the 2009 influenza pandemic. We describe the clinical, epidemiological, and virological features of the first confirmed human case of swine influenza A(H1N2)v in England in this case report. Following this unusual and unexpected finding, we aimed to determine, through contact tracing and sampling of exposed individuals, whether there had been any human-to-human transmission which led to the infection or any onwards transmission.

## Case detection and description

The United Kingdom Health Security Agency (UKHSA) operates community respiratory virus surveillance with the Royal College of General Practitioners (RCGP) Research and Surveillance Centre (RSC) [1,2]. In November 2023, an influenza A-positive sample from the RCGP surveillance scheme was confirmed as subtype H1N2. The sample was taken from a an 80-year-old individual in northern England who presented with a 4-day history of cough, shortness of breath and production of green sputum. The individual had been vaccinated 6 weeks prior with 2023/24 seasonal influenza vaccine and was treated with oral antibiotic (amoxicillin) but did not require hospitalisation. The illness gradually resolved over the following week.

Initial testing of the respiratory sample indicated an influenza A infection with high quantification cycle value (Cq = 33), close to the normal limit of detection (Cq = 40). The subtype of the sample was not identified by RT-PCR for detection of seasonal influenza A (H1N1)pdm09 or H3, suggesting the possibility of an unusual virus variant. Whole genome sequence analysis undertaken using the Illumina platform (Illumina, San Diego, United States) was consistent with a swine H1N2 virus infection belonging to clade 1B.1.1 [3,4].

## Virological investigations and genomic analysis

**Figure 1:**
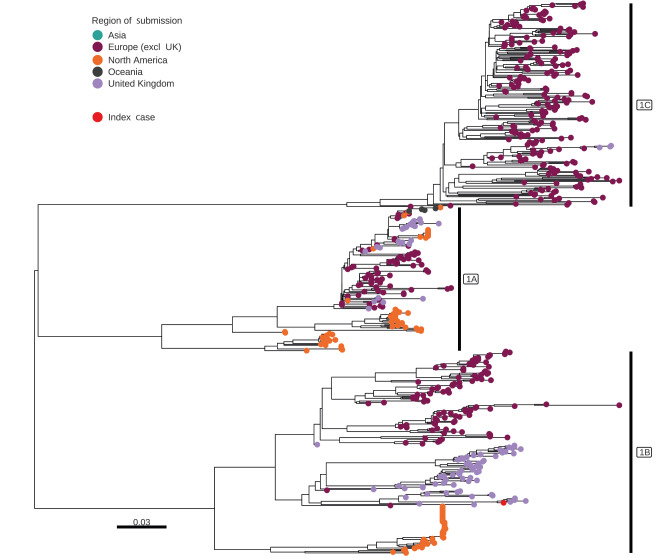
Phylogeny of global influenza A (H1) swine sequence data and the viral sequence of the case in this report, United Kingdom, November 2023

**Figure 2:**
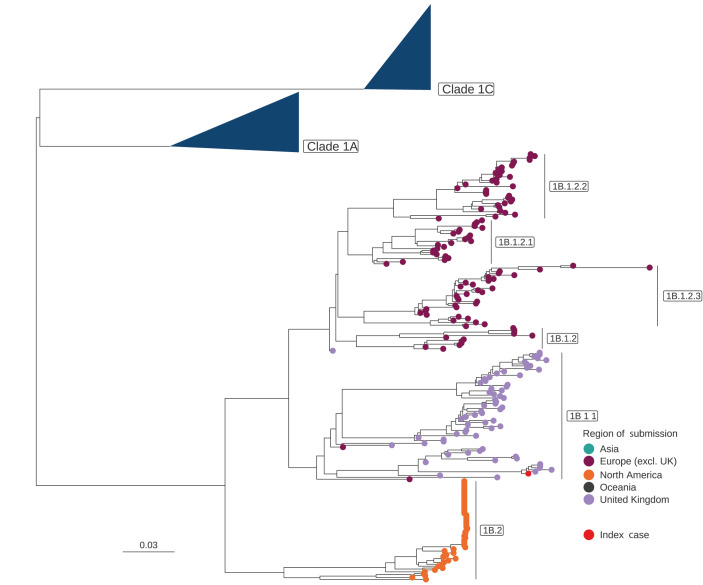
Phylogeny of influenza A(H1) 1B swine sequence data and the viral sequence of the case in this report, United Kingdom, November 2023

We attempted virus isolation from the residual clinical material using MDCK and MDCK-Siat cells, but no virus was recovered. We compared the sequences from the human clinical material (GISAID: EPI_ISL_18548251 and NCBI GenBank: SUB140.02372) with influenza A sequences from pigs in Great Britain (GB) and Europe via the Animal and Plant Agency (APHA) and the World Health Organisation (WHO) Collaborating Centre for Influenza Research and Response at the Francis Crick Institute (FCI). Genomes from contemporary swine influenza cases across GB have been made available on GISAID and are listed in the Supplement. Genomic assessments were undertaken using the complete genome from the human case. The haemagglutinin (HA) gene sequence from the human case was closely related to viruses detected in swine in GB in 2022 and 2023. The contemporary swine influenza viruses and the variant clustered genetically within the 1B.1.1 HA lineage. Different genetic subgroups of the 1B.1 lineage circulate in different parts of Europe but, to date, the 1B.1.1 lineage, derived from the introduction of seasonal human influenza in the 1980s, has only been detected in pigs in GB (Figure 1, Figure 2). The neuraminidase (N2) and the internal gene segments all demonstrated a very close relationship to contemporary H1N2 swine influenza A viruses circulating in GB with no evidence of a new reassortment. Nucleotide or amino acid differences between the human strain and contemporary swine viruses or designated nearest surrogate virus are shown in Supplementary Tables S1 and S2, respectively. In summary, the viral genome from the human case clustered closely with contemporary swine viruses from the surrounding region. 

UK: United Kingdom.

The phylogeny is labelled by the three major first order H1 lineages. Tip points are coloured by the region in which they were collected. Sequence data were collated from GISAID and NCBI virus, aligned with MAFFT v7.487 and trimmed manually in Aliview. Phylogeny was inferred using the maximum likelihood approach in IQ-Tree v2.1.4 with ModelFinder and 1,000 ultrafast bootstraps. Phylogeny was visualised using R version 4.1.1 with the libraries ggplot2, ggtree, ape and tidytree.

UK: United Kingdom.

The influenza A(H1) 1A and 1C lineages have been collapsed and 1B second and third order lineages are labelled. Tip points are coloured by region in which they were sampled. The UK index case is indicated by the red tip point. Sequence data were collated from GISAID and NCBI virus, aligned with MAFFT v7.487 and trimmed manually in Aliview. Phylogeny was inferred using the maximum likelihood approach in IQ-Tree v2.1.4 with ModelFinder and 1,000 ultrafast bootstraps. Phylogeny was visualised using R version 4.1.1 with the libraries ggplot2, ggtree, ape and tidytree.

Several mutations were identified from comparisons between human-derived A(H1N2)v genome and the recent 1B.1.1 swine influenza A virus genomes. For the amino acid differences between the human strain and the designated surrogate swine virus we refer to Supplementary Table S2. There were no obvious features of concern in the mutations seen in the human viral sequence compared with the swine viral genomes, although some differences are of unknown significance.

## Antigenic analysis

A swine influenza A(H1N2) virus from 2023 (GISAID accession number ID: EPI_ISL_18543639) was designated as the nearest surrogate virus to our human case virus and used for phenotypic assessments and diagnostic assurance work. The antigenic properties of our influenza A(H1N2)v virus have not been determined, as there was no virus isolate, but antisera raised in ferrets to closely related swine influenza viruses, including the designated surrogate virus EPI_ISL_18543639, showed poor cross-reactivity in haemagglutination inhibition assays using human seasonal vaccine viruses recommended for use in the 1980s, probably as a result of independent antigenic drift in swine. Assessment of recent 1B.1.1 viruses from swine using human sera from a cohort vaccinated in 2022 showed no immunological recognition of these 1B.1.1 swine influenza A viruses. A ferret antiserum raised against A/Wisconsin/588/2019 (the 2022/23 seasonal influenza vaccine used in the Hong Kong cohort) also did not immunologically recognise these 1B.1.1 lineage viruses [5] (Nicola Lewis, personal communication, December 2023). In addition, a small panel of paired human GB sera (n = 20), taken pre- and post-vaccination with the current 2023/24 northern hemisphere seasonal influenza vaccine, showed no reactivity to the designated surrogate H1N2v. The detailed results for the 20 sera are made available in Supplementary Table S3.

## Public health investigations and control measures

The public health response was delivered through a multiagency incident management team, with veterinary and human health sectors fully represented [6]. 

Comprehensive backward contact tracing from the index case identified that, although the individual lived in a region of the country with a high density of pigs, there was no animal contact in the history of the case or their household contact (no pigs, no pets and no contact with environments that were obviously contaminated by animals). A household contact had also been unwell around the same time but had not sought medical attention or been swabbed. For both individuals, the illness resolved over a period of 7–10 days. The household contact who developed clinical disease was therefore classified as a probable case. 

On forward contact tracing, four additional individuals were identified, three of whom were asymptomatic following potential exposure (to the index case) but had swabs taken on a precautionary basis. None of them tested positive for influenza A. The fourth contact was a healthcare worker (HCW) who had seen the index case at an outpatient appointment on the day after illness onset regarding an unrelated condition. The HCW became unwell with a mild respiratory illness involving a runny nose and cough 9 days after exposure to the index case and was designated as a further probable case. The illness was considered minor and resolved fully without complications. A swab taken on 26 November (10 days after symptom onset) tested negative for influenza A but positive for rhinovirus.

Overall, we identified seven individuals, of whom two were designated as probable cases. Secondary contact tracing was initiated around the HCW probable case on a precautionary basis, and 198 contacts (38) with symptoms compatible with influenza-like illness (ILI) were identified. For 149 of them, PCR tests have been requested, with 88 negative results for influenza A, one positive for H1N1 and 60 results pending or lost to follow-up at 17 January 2024. 

General practices participating in sentinel swabbing in the surrounding area were asked to increase swabbing of patients presenting with acute respiratory infection, and more general practices have been recruited to the established sentinel respiratory virus surveillance scheme. There has been widespread circulation of influenza, respiratory syncytial virus and rhinovirus between November and December 2023, but there have been no unusual spikes of respiratory activity in and around the affected areas [7]. No further cases of influenza A(H1N2)v have been identified since the time of incident termination on 22 December 2023, through enhanced community-based surveillance and analysis of influenza A-positive samples from hospitals in the affected areas [6,7].

## Discussion

Influenza A(H1N1)pdm09 (1A.3.3.2) and avian-like influenza A (1C) (H1N1) swine viruses cocirculate with H1N2 viruses in swine populations across GB. These H1N2 viruses were detected as having emerged in GB swine in the early 1990s, probably resulting from a reverse zoonotic event involving human seasonal viruses which then persisted in pigs following multiple reassortment events with endemic swine viruses [8]. Between 2014 and 2023, H1N2 viruses accounted for ca 65% of virus detections in pig herds (Animal and Plant Health Agency, data not shown).

Zoonotic transmission of swine influenza A to humans can result in an ILI similar to human seasonal influenza. Since 2010, more than 500 cases of infection with variant influenza viruses of swine origin have occurred in the United States, the majority of which were H3N2v, with a much smaller number of H1N1v or H1N2v viruses [9]. Human infections with H1v (1C) have occurred sporadically in Asia and Europe; the most recent was a single detection of an H1N1v virus in the Netherlands in September 2023 [10], also with no known contact with pigs and no onward transmission.

The H1 swine influenza viruses are antigenically diverse, with origins linking back to 1918 and classical swine influenza precursors (1A) including the H1N1pdm09 (1A.3.3.2), pre-2009 seasonal H1N2 (1B) and Eurasian avian-like H1N1(1C). All have drifted antigenically from either their human seasonal or an avian ancestor. Serological testing of human populations vaccinated with seasonal influenza vaccines have demonstrated that the 1B and 1C HA lineage H1v viruses show very little to no cross-reactivity, indicating a lack of immunity even in a vaccinated population, as demonstrated in this case [5] (Nicola Lewis, personal communication, December 2023).

Viral factors permitting swine-to-human zoonotic infection remain largely undefined [11]. It is currently not possible to predict which of the clades of swine influenza A viruses causes human infections or has a propensity for sustained human-to-human transmission, leading to a global pandemic such as occurred in 2009. The risk of variant infection of humans relates to the risk of mucosal contamination and aerosols at animal–human interfaces such as, but not limited to, farm environments, live animal markets and agricultural fairs, along with the nature of the variant virus and unknown virus and host characteristics.

To ensure preparedness, WHO Human seasonal influenza Vaccine Composition Meetings also include the review of swine candidate vaccine strains (CVVs), updated regularly to be representative of circulating swine strains [5]. Based on the assessments undertaken following this case detection, currently available CVVs for H1 1B swine influenza viruses are unlikely to afford protection against the H1 1B.1.1 swine influenza A viruses detected in GB.

## Conclusion

The identification of this variant infection occurred due to an unusual pattern of reactivity in laboratory diagnostic assays. Work is underway with the Medicines and Regulatory Agency, to assess the detection capability of-commercial and non-commercial platforms used in the United Kingdom. Gaining assurance about the ability of available clinical diagnostic assays to detect swine variant influenza viruses is a complex task, with the necessity to develop rapid response arrangements for clinical diagnostic assurance when new influenza variants are detected in humans, and to develop external quality assessment schemes with a broad range of zoonotic viruses.

## Supplementary Data

161000 Supplement
