# Authors’ correction for Euro Surveill. 2024;29(3)

**DOI:** 10.2807/1560-7917.ES.2024.29.4.250125c

**Published:** 2024-01-25

**Authors:** Eurosurveillance editorial team

**Affiliations:** 1 European Centre for Disease Prevention and Control (ECDC), Stockholm, Sweden

In the article entitled ‘A case of swine influenza A(H1N2)v in England, November 2023’ by Cogdale et al., published on 18 January 2024, a funding source was corrected in the Funding statement at the request of the authors: “funded by the NIH Centres of Excellence in Influenza Research and Response programme: AA No. 75N93019R00028 through a collaboration between USDA-ARS, APHA, the Royal Veterinary College and the Francis Crick Institute” was changed to “funded by the NIH Centres of Excellence in Influenza Research and Response programme: AA No. 75N93021C00015 through a collaboration between Penn-CEIRR, USDA-ARS, APHA, the Royal Veterinary College and the Francis Crick Institute”. This correction was made on 24 January 2024.

