# Supplementary material for: A case of swine influenza A(H1N2)v in England, November 2023
Source: Euro Surveill. 2024 Jan 18;29(3):2400002. doi: 10.2807/1560-7917.ES.2024.29.3.2400002 (PMC10797662; doi:10.2807/1560-7917.ES.2024.29.3.2400002)
Supplement: Supplement [file 24-00002_ZAMBON_Supplement.pdf]

"This supplementary material is hosted by *Eurosurveillance* as supporting information alongside the article [A case of swine influenza (H1N2)v in England, November 2023], on behalf of the authors, who remain responsible for the accuracy and appropriateness of the content. The same standards for ethics, copyright, attributions and permissions as for the article apply. Supplements are not edited by *Eurosurveillance* and the journal is not responsible for the maintenance of any links or email addresses provided therein."

Supplementary description of sentinel surveillance

Through this system, 170 general practitioner (GP) practices across England swab patients presenting with new episodes of acute respiratory illness (ARI) (1). The protocol undertaken for winter season 23/24 is available at (2). Samples are routinely analysed for influenza A, B, RSV, hMPV, seasonal coronaviruses (OC43, 229E, HKU1, NL63), SARS CoV 2, adenovirus, rhinovirus and enteroviruses. Influenza A and B positive samples are characterised to determine the virus subtype and where possible, analysed by whole genome sequencing (WGS) to provide insights about circulating virus diversity.

Supplementary Information 1. GISAID accession numbers of GB swine sequences

EPI_ISL_18543639, EPI_ISL_18586964, EPI_ISL_18586963, EPI_ISL_18586962, EPI_ISL_18586961, EPI_ISL_18586960, EPI_ISL_18586959, EPI_ISL_18586958, EPI_ISL_18586957, EPI_ISL_18586956, EPI_ISL_18586955, EPI_ISL_18586954, EPI_ISL_18586953, EPI_ISL_18586952, EPI_ISL_18586951, EPI_ISL_18586950

Supplementary Table 1. Nucleotide identity for human gene segments against contemporary swine influenza viruses

Segment	Max % ID	Min % ID	Closest swine strain	EPI ID
PB2	98.76	82.49	A/swine/England/123383/2023	EPI_ISL_18586956
PB1	98.99	80.79	A/swine/England/123045/2023	EPI_ISL_18543639
PA	98.84	82.26	A/swine/England/123383/2023	EPI_ISL_18586956
HA	98.73	53.5	A/swine/England/123383/2023	EPI_ISL_18586956
NP	99.46	81.36	A/swine/England/123383/2023	EPI_ISL_18586956
NA	98.83	50.19	A/swine/England/123383/2023	EPI_ISL_18586956
MP	99.08	86.23	A/swine/England/123045/2023	EPI_ISL_18543639
NS	99.14	77.82	A/swine/England/123383/2023	EPI_ISL_18586956

Supplementary Table 2. Differences by segment between the human case and designated surrogate virus (A/swine/England/123045/2023)

Segment	List of mutations

Seg 1 PB2	K70R, N102X, G103X, G590S, I649V, F694X, L695S, I696X
Seg 2 PB1	V113I, Y431H, N642S
Seg 3 PA	K196R, M261L, V379I, T528S, I542V, N614S
Seg 4 HA	Y10C, Q68H, F112L, M133I, K158E, S159R, Y278F, I289M, I341V, V362I, H370Q, S468G, T511N
Seg 5 NP	No amino acid substitutions
Seg 6 NA	Y42C, S43G, L58V, I71T, K77N, Y155H, G286S, I302V, D346N, L372M, I418T
Seg 7 MP	H26Q, A227T
Seg 8 NS	C60F, G71E, S91T, K104M

Supplementary Table 3. To assess potential protection from the current northern hemisphere 2023-24 vaccine, a Haemagglutination Inhibition (HAI) assay was performed with a small panel of paired pre-and post-vaccination samples from Crick volunteers receiving the 2023-24 egg-based vaccine. Egg-propagated IVR-238 A/Victoria/4897/2022 is representative of the H1N1pdm09 component of the 2023/24 vaccine, whereas cell-propagated A/swine/England/045393/2022 is a representative of the 1B.1.1 clade which the H1N2v. HAI carried out using 0.7% Turkey RBC.

Sample	Crick ID	A/swine/England/ 045393/2022	IVR-238 A/Victoria/4897/2022
pre	44	<20	160
post	44	<20	1280
pre	50	<20	1280
post	50	<20	1280
pre	61	<20	1280
post	61	<20	1280
pre	82	<20	1280
post	82	<20	1280
pre	99	<20	1280
post	99	<20	1280
pre	140	<20	1280
post	140	<20	1280
pre	147	<20	160
post	147	<20	320
pre	148	<20	80
post	148	<20	160
pre	150	40	80
post	150	40	160
pre	151	<20	40
post	151	<20	160

pre	158	<20	<20
post	158	<20	<20
pre	166	<20	160
post	166	<20	160
pre	172	<20	40
post	172	<20	160
pre	179	<20	40
post	179	<20	160
pre	183	<20	160
post	183	<20	160
pre	187	<20	160
post	187	<20	<20
pre	190	40	320
post	190	40	640
pre	199	<20	320
post	199	<20	320
pre	201	<20	<20
post	201	<20	160
pre	217	<20	80
post	217	<20	160
A/Victoria ferret		<20	1280
A/Swine ferret		80	<20
